# Developing criteria for a profession to be considered as profession of allied health in Malaysia: a qualitative study from the Malaysian perspective

**DOI:** 10.1186/s12913-024-10569-0

**Published:** 2024-02-02

**Authors:** L Mageswary Lapchmanan, Duratul Ain Hussin, Naji Arafat Mahat, Aik Hao Ng, Nurul Huda Bani, Salina Hisham, Wai Siew Teh, Mohd Azmarul A Aziz, Saravanakumar Maniam, Pauzilah Dollah, Nur Atiqah Hasbullah, Salini Manimaran, Hazirah Hassan, Farina Zulkernain

**Affiliations:** 1 Allied Health Sciences Division, Ministry of Health Malaysia, Putrajaya, Malaysia; 2 Department of Chemistry, Faculty of Science, Universiti Teknologi Malaysia, Johor Bahru, Johor, Malaysia; 3 Centre for Sustainable Nanomaterials, Ibnu Sina Institute for Scientific and Industrial Research, Universiti Teknologi Malaysia, Johor Bahru, Johor, Malaysia; 4 Centre of Research for Fiqh Forensics and Judiciary, Faculty of Syariah and Law, Universiti Sains Islam Malaysia, Nilai, Negeri Sembilan Malaysia; 5 Faculty of Medicine, Universiti Malaya, Kuala, Lumpur, Malaysia; 6 Audiology Unit, Department of Rehabilitation Medicine, Cheras Rehabilitation Hospital, Kuala Lumpur, Malaysia; 7 Department of Forensic Medicine, Hospital Sultan Idris Shah Serdang, Selangor, Malaysia; 8 Nutrition Division, Ministry of Health Malaysia, Putrajaya, Malaysia; 9 School of Health Sciences, Universiti Sains Malaysia, Kubang Kerian, Kelantan, Malaysia

**Keywords:** Allied health profession act, Criteria for profession of allied Health, Allied health professions, Focus group discussion, Document review analysis, Malaysia

## Abstract

**Background:**

The Malaysian Allied Health Profession Act (Act 774) regulates the practice of allied health practitioners in Malaysia, with two described professions *viz*. allied health profession (AHP) and profession of allied health (PAH). While AHPs have been clearly identified by the law, comprehensive implementation of the act requires development of specific criteria in defining any profession as PAH in the Malaysian context. Hence, the research aims to explore and identify the criteria for defining such professions for healthcare policy direction in Malaysia.

**Methods:**

This research utilised two methods of qualitative research (document review and focus group discussions (FGDs) involving 25 participants from four stakeholders (higher education providers, employers, associations and regulatory bodies). Both deductive and inductive thematic content analysis were used to explore, develop and define emergent codes, examined along with existing knowledge on the subject matter.

**Results:**

Sixteen codes emerged from the FGDs, with risk of harm, set of competency and skills, formal qualification, defined scope of practice, relevant training and professional working within the healthcare team being the six most frequent codes. The frequencies for these six codes were 62, 46, 40, 37, 36 and 18, correspondingly. The risk of harm towards patients was directly or indirectly involved with patient handling and also relates to the potential harms that may implicate the practitioners themselves in performing their responsibilities as the important criterion highlighted in the present research, followed by set of competency and skills.

**Conclusions:**

For defining the PAH in Malaysia, the emerged criteria appear interrelated and co-exist *in milieu,* especially for the risk of harm and set of competency and skills, with no single criterion that can define PAH fully. Hence, the integration of all the empirically identified criteria must be considered to adequately define the PAH. As such, the findings must be duly considered by policymakers in performing suitable consolidation of healthcare governance to formulate the appropriate regulations and policies for promoting the enhanced framework of allied health practitioners in Malaysia.

**Supplementary Information:**

The online version contains supplementary material available at 10.1186/s12913-024-10569-0.

## Background

Reforms in the healthcare systems have been widely advocated by developed countries [1, 2], in tandem with a myriad of evolving factors such as the prevalence of chronic non-communicable diseases, rapidly aging populations and limited skilled healthcare professionals [3]. Moreover, the situation is further exacerbated by higher consumer expectations as well as the ever-rising cost of treatment and containment throughout the world [4, 5] including Malaysia [6]. In this context, the involvements of allied health professionals within the healthcare framework appear relevant to provide sufficient services to the public at large [7]. Pertinently, the term allied health professionals alone has been prevailingly used in the literature (e.g. Bissett et al., 2021 [8]; Seaton et al., 2021 [9]) as well as in jurisdictions in countries like the United States of America (USA) [10], the United Kingdom (UK) [11], Australia [12] and Singapore [13]. These countries have taken several approaches in defining allied health professions and eventually concluded with a general description of allied health professionals instead.

As for Malaysia, a specific act for allied health, The Malaysian Allied Health Profession Act (Act 774) was drafted in 2008, gazette on February 18, 2016, and enforced on July 1, 2020. Act 774 has been enacted to enable the establishment of the Malaysian Allied Health Professions Council (MAHPC), the registration of allied health practitioners and persons carrying out allied health activities, as well as regulating the professions, practice, and other related matters. Interestingly, unlike other countries, Act 774 that regulates the practice of allied health in Malaysia provides two separate descriptions of such professions *viz.* allied health profession (AHP) and profession of allied health (PAH). The PAH is considered as the outer perimeter, while the AHP is the directly regulated subset component of the healthcare framework by the MAHPC. Hence, the flexibility gives the law the dynamics to comprehensively embrace suitable reforms in the healthcare workforce as well as other aspects of global healthcare systems. The law describes the 16 AHPs as the “profession of allied health specified in the Second Schedule and any activity relating to allied health prescribed by regulations under Section 11” (Act 774). The professions include audiologist, dietitian, entomologist (public health), physiotherapist, medical physicist, nutritionist, clinical psychologist, diagnostic radiographer, medical laboratory scientist, occupational therapist, speech therapist, radiation therapist, medical laboratory technologist, dental technologist, environmental health officer, and health education officer. Under the law (Part 3, Section 10), amendments can be made to the Second Schedule, considering the expansion of relevant healthcare professions in the country.

On the other hand, PAH means “any profession which has a direct or an indirect effect on patient care, or on the health of an individual or the population” (Act 774). Nevertheless, the law does not provide any criteria for identifying professions that can be considered as PAH nor that it specifies individual professions. While a rigid definition may be too restrictive, a vague description may result in confusion at organisational levels [14], including that for PAH. Therefore, performing a specific study for addressing both aspects would prove useful in formulating a suitable regulation that is auxiliary to the law (Act 774) for its implementation in Malaysia. The establishment of a suitable regulation that would strengthen the implementation of Act 774 proves timely and paramount in at least two contextual situations. The first relates to safeguarding the interest of the patients and/or clients as well as second, preventing unnecessary regulatory burdens within the healthcare framework. This is particularly pertinent considering the ever-expanding fields of allied health [15], the inter-relatedness of services provided [9], and the fact that there are professions with overlapping functions. Adding to the confusion, discrepancies in job titles and scopes of work (especially between the public and private sectors) further necessitate the establishment of such a regulation (Allied Health Sciences Division: Review of the Allied Health Professions Act 2016, unpublished).

Hence, this present research that aimed to provide the criteria for professions to be considered as PAH in Malaysia in the healthcare framework, through qualitative methods involving various stakeholders, merits consideration. The data would prove useful in understanding the status of the professions, in view of formulating suitable regulations that would complement the implementation of Act 774 in Malaysia.

## Methods

This research utilised two different qualitative methods (*viz*. document review analysis and Focus Group Discussions, FGDs) for ensuring adequate triangulation of the findings [16] in exploring the criteria of PAH in Malaysia. Ethical approval was obtained from the Medical Research and Ethics Committee (MREC), Ministry of Health Malaysia (MOH) (NMRR-20-3181-57869), prior to the commencement of the research. Upon providing an adequate explanation of the project, FGDs participants were required to sign the informed consent document.

### Document reviews

We reviewed existing documents in Malaysia and other Southeast Asian countries, as well as in developed countries (such as Australia, the UK and the USA) during April to June 2021 prior to performing the FGDs. This method is required in obtaining the scoping of the FGDs including development of the FGDs questions and triangulation of findings. The documents included were Act 774, concept papers, books and official brochures; program handbooks by higher education providers (HEPs); organisational and institutional reports; authentic official websites (HEPs, association, employers and regulatory bodies) and various other public records related to the study aims. Table 1 represents the types of documents included in the document review and the information gathered.

**Table 1:** Main findings from documents identified for the suitable criteria of PAH

**No.**	**Source Documents**	**Main Findings**	**Key Themes**
**1**	**Higher Education Providers** Official brochures, program handbooks, authentic official websites (MQF 2.0 and Framing Malaysian Higher Education 4.0: Future-Proof Talents) Malaysian Qualification Agency (MQA) has released the latest version of Malaysian Qualification Framework version 2.0 (MQF 2.0). **Public Institutions** Universiti Kebangsaan Malaysia (UKM), Universiti Putra Malaysia, Universiti Malaya(UM), International Islamic University Malaysia (IIUM), Universiti Sultan Zainal Abidin (UniSZA), Universiti Teknologi MARA (UiTM) **Private Institutions** KPJ Healthcare University College (KPJUC), MAHSA, SEGi University, UCSI University, Management & Science University (MSU)	**Faculties with Allied Health** Science & Technology, Health Science, Social Sciences & Humanities, Education, Medicine and Health Sciences, Food Science & Technology, Educational Studies, Biotechnology & Biomolecular, Education, Islamic Revealed Knowledge and Human Sciences, Allied Health Sciences, Bioresources & Food Industry, Applied Social Sciences, Sports Science & Recreation, Health Sciences, Medicine, Business & Management Medicine, Bioscience & Nursing, Dentistry, Health Sciences, Social Sciences & Liberal Arts, Pharmaceutical Sciences, Medicine & Health Sciences, Applied Sciences, Health & Life Sciences, Education & Social Sciences **List of Courses related to Allied Health** Biochemistry, Bioinformatics, Food Science & Nutrition, Food Science with Business Management, Genetics, Nuclear Science, Physics, Audiology, Biomedical Science, Clinical Psychology, Diagnostics Imaging & Radiotherapy, Dietetics, Environmental Health & Industrial Safety, Forensic Science, Occupational Therapy, Optometry & Vision Science, Physiotherapy, Speech Science, Developmental Science, Social Work, Special Education, Biomedical Sciences, Dietetic, Nutrition & Community Health, Environmental & Occupational Health, Food Science & Technology, Food Science & Management, Food Studies, Physical Education, Guidance Counselling, Biotechnology, Molecular Biology, Counselling, Educational Psychology, Special Education, Professional Counselling, Physical and Health Education, Microbiology & Molecular Genetics, Biomedical Science, Guidance & Counselling, Speech-Language Pathology, Medical Imaging, Biomedical Science, Radiography, Medical laboratory Technology, Medical Imaging, Biomedical Science, Nutrition Science, Medical & Health Sciences, Occupational & Environmental Health, Food Technology, Halal Food Development, Biology & Biochemistry, Food Processing, Social Work & Counselling, Food Science & Technology, Medical Laboratory Technology, Medical Imaging, Environmental Health, Environmental Health & Safety, Physiology, Health Promotion & Education, Sports Science, Health & Fitness, Physical & Health Education	Standard of Training and Qualification
**2**	**Employers with Allied Health services** authentic official websites **(Health Facts MOH, 2021)** Pantai Hospital* (Ampang branch) Naluri Hidup Sdn. Bhd., Hospital Pusrawi Sdn. Bhd., KPJ Ipoh Specialist Hospital, Sunway Medical Centre Velocity, Gleneagles Hospital Penang, Life Care, Regen Rehab Hospital, Daehan Rehabilitation, Hospital Putrajaya, WeCare Allied Health Center	**Services related to Allied Health** Face-to-face, conventional manner while only one provide services digitally. Common functions and services of a hospital (diagnostic, curative, rehabilitation) Health screening services, Corporate Wellness Programs, mental health, Chronic diet-related diseases screening, Digital health coaching team, Food & weight/blood pressure management, Monitoring tools, Extended Care Services, customized rehabilitative programs for various neurological and orthopedic disorders **Personnel providing Allied Health services** clinical lab technicians, physiotherapists, occupational therapists, speech therapists, exercise therapy, radiographers, dietitian, cardiovascular technologist, clinical psychologists, mental health counsellor, fitness coaches, optometrists, audiologists, pain specialist, physical therapist, speech language therapist, traditional Chinese medicine	Industries and Health Facilities Sectors; Non-standardization in Nomenclature
**3**	**Association with Allied Health Professions-**authentic official websites Malaysian Dietitians’ Association (MDA), British Dietetic Association (BDA), Academy of Nutrition & Dietetics (formerly American Dietetic Association (ADA), Singapore Nutrition and Dietetics Association (SNDA), Malaysian National Society of Audiologists (MANSA), British Society of Audiology (BSA), Malaysian Association of Clinical Biochemist (MACB), American Society for Biochemistry and Molecular Biology (ASBMB), Royal College of Podiatry, Malaysian Society of Clinical Psychology (MSCP), Australian Clinical Psychology Association (ACPA), Malaysian Music Therapy Association (MMTA), Persatuan Prostetik dan Ortotik Malaysia (PPOM)	**Professions related to Allied Health** dietitian, nutritionist, audiologist, biochemist, podiatrist, clinical psychologist, music therapist, as well as orthotist and prosthetist **Functions of association** Associations serve as a platform to advance the profession as well as increasing and maintaining the professionalism of its members. Other functions include to promote collaboration with other health professionals and to protect its member by providing insurance and legal coverage. Among the memberships offered are ordinary/full membership, student membership, affiliate and honorary membership. Some professions offered more categories of membership, such as non-practising membership, retired membership, corporate/business membership as well as overseas membership.	Code of Professional Ethics
**4**	**Healthcare Professionals’ Acts and Regulations** Legislation documents retrieved online Dental Act 2018, Act 804 Optical Act 1991, Act 469 Registration of Pharmacists Act 2951, Act 371, Nurses Act 1950 Medical Act 1971, Act 50, Traditional and Complementary Medicine Act 2016, Food Analysts Act 2011	The main function of regulatory body across all acts are registration for practitioners and regulating the practices (standards of practice, ethics and professional conduct, scope of practice) and recognise qualifications. In disciplinary inquiry and punishments. In addition, the Food Analyst Act also function to issue guidelines to specify procedures and test methods for food. All acts enforced with at least two other acts which indirectly related to own act such as Dental Act 2018 and Medical Act with Private Healthcare Facilities and Services Act 1998. On top of those acts, there are additional regulatory framework documents that complements the legislation of the acts. Council members are respective profession members who must be Malaysian, residing in Malaysia, affiliated with institution from both public and private sector. The number of council members are odd, proportionate to the size of the professions the act is regulating.	Risk of Harms; Regulations for PAH

Iterative thematic analysis was used by literally interpreting the actual words/sentences published in the documents, followed by summary of the findings in a thematic table.

### Focus group discussions

#### Participants

In view of selecting participants with adequate proficiency in the subject matter as well as voluntarily sharing their experiences and opinions “in an articulate, expressive, reflective manner” [17], participants who fulfil the inclusion criteria representing varying stakeholders were recruited. For participants from HEPs, the inclusion criteria were: (a) academics with experience of at least 15 years and (b) with deep understanding on the medical or health sciences or related programs. In addition, they must also (c) have held senior leadership positions for at least five years in curriculum and program development as well as academic-related matters to the faculty, and/or (d) the appointed representative with an equivalent responsibility. As for employers, the inclusion criteria were: (a) individuals holding the position of senior human resource (HR) manager, head of recruitment or chief HR officer or higher, (b) involve in the HR management including HR strategy and planning, recruitment, selection and induction, training, development and career planning, and performance appraisal (c) with at least 10 years of working experience in the healthcare organisation or institution. For representatives of health science related associations, the inclusion criteria were: individuals (a) holding executive positions such as president, vice president or secretary of the association, (b) having the minimum qualification of a diploma in health sciences (minimum candidature for graduation of two years), and (c) with at least five years of membership in that association. The final inclusion criterion for representatives of regulatory bodies was individuals having at least five years of experience in the executive regulatory body. One exclusion criterion applicable to all the categories of participants was the fact that they have served as officers at AHSD, MOH.

#### The FGD protocol

A FGD protocol was developed consisted of stakeholders group, FGDs composition and FGD questions (Supplementary 1). A pilot FGD was conducted in July 2021 for assessing the appropriateness of the developed protocol and questions which later were improved for the actual FGDs. Participants with similar background to the actual FGDs were included, so that the finalised protocol can be used in the actual FGDs. The FGD questions were derived from the document review analysis and consensually determined through two series of workshops with the researchers. The questions consists of general and specific questions for each stakeholder groups which are homogenous and mixed group (heterogeneous) ranging from six to eight questions per group.

#### The execution of FGDs

Due to the restriction of movement control order following COVID-19 pandemic in Malaysia, the FGDs were conducted *via* a virtual Webex by Cisco platform (audio-recorded). The FGDs were performed in English between August 2021-September 2021, consisted of four homogenous groups (HEPs, employers, associations, and regulators) with the remaining two as heterogeneous (mixture of the four stakeholder’s groups). Each homogeneous FGD was facilitated by a moderator and a note taker, while for the heterogeneous groups there was a co-moderator. The appointed moderators were individuals with general knowledge on allied health and well-versed in general regulations relating to healthcare practitioners with no vested interest in the research. Each moderator was required to encourage the discussion as well as to provide equal opportunities for all participants to express their opinions on the topic, without exerting his/her own opinion that may influence the participants when responding to the questions. The length of each FGD was between 1.5 - 2.5 hours.

Each FGD was verbatim transcribed manually by NAH, and grammatical errors made by the participants were corrected as necessary for better comprehension. In cases whereby a participant used mixed languages (e.g. English and Malay), the phrases in Malay were translated into English by NAH who is native speaker with high proficiency in English. The initial transcripts were submitted to the respective participants for them to verify their answers, translation and corrected grammatical errors without adding any new statements. Subsequently, changes were made (if any) to the initial transcription according to the revised version, and rendered anonymous by assigning a pseudonym to each participant. A third person/party name was removed from the transcript to maintain its confidentiality.

Thematic analysis of the data was made in accordance to Vaismoradi M et al (2013) recommendation [18]. Both the deductive and inductive approaches were used in the analysis taking into consideration the existing knowledge from the findings of document review and the structural elements that disclosed the participants’ critical perspective, resolutions and assessments. Inducted approach was applied later in the refinement of codings for allowing the inclusion of unexpected themes that may emerge during data collection, respectively.

The transcripts were read repetitively before pre-coding was applied based on the document reviews themes, using ATLAS.ti version 9. Discussions were held and consensus among researchers are achieved to resolve discrepancies in the coding.

Subsequently refinement of the coding was performed by DAH and any newly identified codes and themes (if any) were made. The results were shared in the updated thematic code-book with other researchers and discussed until final consensus was achieved.

## Results

### Demographic data and basic information of FGDs participants

Table 2 represents the demographic data for participants recruited for the FGDs, representing the important stakeholders (*viz.* HEPs, employers, associations and regulatory bodies). A total of 25 participants were included in the FGDs with each FGDs comprised of four or five participants. It was observed that 11 participants have PhD degree while eight have master’s degree and six with bachelor degree. Majority of the participants were Malays (17 participants), followed by six Chinese and two Indians.

**Table 2:** Demographic breakdown of participants recruited for the FGDs

**Indicators**	**HEPs**^a^	**Employer**^b^	**Association**^c^	**Regulatory Body**^d^	**Total Participants**
**Gender**					
Male	3	1	2	2	8
Female	4	5	4	4	17
**Age (years)**					
<30	-	-	1	-	1
31-40	1	1	2	1	5
41-50	3	2	2	4	11
51-60	3	3	-	1	7
>60	-	-	1	-	1
**Ethnicity**					
Malay	6	5	2	4	17
Chinese	1	1	4	-	6
India	-	-	-	2	2
**Highest education level**					
Degree	-	2	1	3	6
Master	-	4	2	2	8
PhD	7	-	3	1	11

^a^Participants are from 4 public universities and 3 private universities

^b^Participants are from 2 public employers and 4 private employers

^c^Participants are representative from Malaysian Music Therapy Association (MMTA), Malaysian Association of Speech-Language & Hearing (MASH), Genetic Counselling Society Malaysia (GSM), Nutrition Society of Malaysia (NSM) and International Society for Prosthetics and Orthotics (ISPO)-Malaysia Chapter

^d^Participants are representative from the Traditional and Complimentary Medicine Council (T&CM Council), Pharmacy Board Malaysia (PBM), Malaysian Optical Council (MOC), Malaysian Dental Council (MDC), Medical Assistant Board (MAB) and Malaysian Nursing Board (MNB)

The representative of six HEPs are those from both public and private institutions including health institutions with embedded training programs. A total of six associations representatives were included in the FGDs. These associations set up the minimum standard for professional practice, providing continuous education development and promoting research and evidence-based practices. As for the regulatory bodies, their main functions include registering the practitioners and issuing practicing certificates (temporary, provisional or full registration).

### Document review analysis

The key themes derived from the document reviews are described in Table 1.

#### Standard of training and qualification

The most commonly offered allied health programs are physiotherapy, dietetics, nutrition and medical laboratory. In view of curriculum development for HEPs in Malaysia, the Malaysian Qualification Agency (MQA) through the Malaysian Qualification Framework version 2.0 (MQF 2.0) focuses on the relevant graduate attributes, program educational outcomes, program learning outcomes, course learning outcomes and constructive alignment. In this regard, involvement of stakeholders is an integral aspect for curriculum development by the HEPs to ensure that the curriculum is relevant and it covers important aspects of practice including risk management. Acknowledging the importance of embracing the fourth industrial revolution in the higher education sector, the Ministry of Higher Education (MOHE) has published an important document depicting the aspirations, objectives, and approach to produce graduates that are capable to deal with artificial intelligence, digitisation, automation, and Internet of Things (IoT) in daily experience. The document (Framing Malaysian Higher Education 4.0: Future-Proof Talents) has become one of the sources of reference in curriculum development including for those of medicine and health sciences programs.

#### Industries and health facilities sectors

Table 3 summarises the localities of PAH employed by different ministries in Malaysia as of 2020. The MOH remains the primary employer for the public sector followed by several other ministries (MOHE, Ministry of Defense, Ministry of Education, Ministry of Women, Family and Community Development, Ministry of Youth and Sports and Ministry of Science, Technology and Innovation). Having considered the different government entities that were involved, it can be seen that the name of profession as well as the job scope remains generally similar.

**Table 3:** PAH employment locations in public sector by different ministries in 2020

Ministry	PAH employment locations
Ministry of Health	135 hospitals
	11 special medical institutions
	2890 health clinics
	257 community clinics
	6 research institutions
	19 training institutes
Ministry of Higher Education	9 teaching hospitals
	teaching and support staff at faculties of medicine, allied health and sciences
Ministry of Defense	hospitals and health facilities
Ministry of Women, Family and Community Development	welfare facilities
Ministry of Youth and Sports^a^	National Sports Institute and National Sports Council
Ministry of Education^a^	sports schools
	special needs schools
Ministry of Science, Technology and Innovation	research facilities

^a^specialised PAHs such as physiotherapist and nutritionist

It can be seen that PAHs were involved in three main sub-sectors at the private sectors *viz.* privately-owned hospitals/facilities, rehabilitation centres as well as medical laboratories, whereby the majority of them provide services in face-to-face sessions as well as *via* digital means. Those that provide services in face-to-face sessions are at (a) private hospitals providing the full range of services from prevention to curative and rehabilitation, (b) institutions that focus on rehabilitation alone and (c) facilities that focus on specific health issues (i.e. mental and developmental disorders). Specifically, for private hospitals, their services included health screening, clinical and diagnostic services, dietetic and food services, and rehabilitation services (including physical and mental health). Among the professional team, professions such as dietitian, physiotherapist, occupational therapist and speech-language therapist were the commonly highlighted ones. Despite their pivotal roles in healthcare management and treatment, these private healthcare providers rarely mentioned laboratory-based personnel in published documents.

#### Non-standardisation in nomenclature

Discrepancies prevailed between the government and private sectors as well as with that specified in Act 774. For example, the profession of Medical Rehabilitation Officer (Speech) designated in the MOH is known as speech-language therapist in Act 774, consistent with the International Standard Classification of Occupation (ISCO-08). The same profession is interchangeably known as speech therapist, speech pathologist and speech-language pathologist in the private sector although the scope of work appears similar with that of the government sector. Echoing to the global adaptation of the fourth industrial revolution that integrated human capital development, artificial intelligence, digitisation, automation, and IoT, transformation in the healthcare services in Malaysia has also taken place. This is because adaptation of such an approach would enable digital innovation in healthcare delivery and solutions, as well as talent development, preparing Malaysia to become an important digital health innovation hub.

#### Risk of harms

PAHs are exposed to multitude of risks of harm in performing their professional tasks. Specific guidelines on occupational safety and health management have been imposed to protect the employees ‘from hazards and its associated risks, the elimination of work-related injuries, disabilities, ill health, diseases, near misses and fatalities’ [19]. This is also consistent with the document titled ‘Occupational safety and health in public health emergencies: a manual for protecting health workers and responders’ published by the World Health Organization (WHO) and International Labour Organization (ILO)(2018) in facing disease outbreaks and other emergencies related to ‘natural disasters, chemical incidents, radiological emergencies and emergencies involving conflicts’, focusing on low resource settings. The key elements of the document revolve around reducing occupational exposures, injury, illness and death, stress and fears, as well as promoting good health and well-being. To achieve such a goal, (a) having the adequate managerial and technical tools as well as strategies for dealing with occupational safety and health hazards and (b) the complete understanding of the different types of emergencies are the central focuses of the document. To legalise this aspect in Malaysia, compliance by employers is mandatory pursuant to national laws and regulations. It requires the establishment of an Occupational Safety and Health Management System (OSHMS) that comprises elements of policy, organising, planning and implementation, as well as evaluation and action for improvement.

#### Code of professional ethics

Associations play role in the defining standards for the practice of the profession, regulatory practices for maintaining professional standards, aims and objectives, communications and the different tier of memberships. While defining the standards for the profession would involve the appropriate training, practice and continuous professional development (CPD) for providing reliable and evidence-based services, regulatory practices relate to protecting the professional interests of the profession and for safeguarding the public at large. As for the aims and objectives, the different health-related associations reviewed appear to share similar approaches and philosophies. They included promoting the professional and ethical practices, its relevance, developing a workforce strategy, collaborating with the various stakeholders and influencing health policy and regulations. In the context of communication, the establishment of scientific journals, organising online courses, webinars, workshops and public engagements were the commonly discovered strategies. Considering the different levels of education and professional training, the reviewed health-related associations had various types of memberships. They included ordinary/full, student, affiliate and honorary memberships. In addition, categories like non-practicing, retired, corporate/business and international memberships were observed in the documents.

#### Regulations for PAH

Established regulatory bodies related to healthcare practitioners in Malaysia are namely Malaysian Nursing Board, Pharmacy Board Malaysia, Malaysian Medical Council, Medical Assistant Board, Malaysian Optical Council, Board of Counsellor, MAHPC, Traditional and Complimentary Medicine Council and Malaysian Dental Council. These regulatory bodies were associated with the Nurses Act 1950 (Act 14), Registration of Pharmacists Act 1951 (Act 371), Medical Act 1971 (Act 50), Medical Assistants Act 1977 (Act 180), Optical Act 1991 (Act 469), Counsellor Act 1998 (Act 580), Act 774, Traditional and Complementary Medicine Act 2016 (Act 775) and Malaysian Dental Act 2018 (Act 804), respectively. Specifically, Act 774 regulates 16 AHPs (Table 4), Act 469 regulates optometrists and opticians and Act 580 regulates counsellors. Other PAH in Malaysia are generally not regulated by profession-specific act while the practice of self-regulation, quasi-regulation or co-regulation for the related professions has not been found in the documents reviewed.

**Table 4:** List of professions regulated in Malaysia, Australia and the UK

Country	Regulation	Professions
Malaysia	Act 774	Audiologist, Clinical Psychologist, Dental Technologist, Diagnostic Radiographer, Dietitian, Entomologist (Public Health), Environmental Health Officer, Health Education Officer, Medical Laboratory Technologist, Medical Physicist, Nutritionist, Occupational Therapist, Physiotherapist, Radiation Therapist, Speech-Language Therapist, and Medical Laboratory Scientists (Biochemist, Biomedical Scientist, Embryologist, Medical Geneticist, Microbiologist and Forensic Science Officer)
Australia	National Registration and Accreditation Scheme (NRAS)	Aboriginal and Torres Strait Islander Health Practitioners, Chinese Medicine Practitioners, Chiropractors, Dental Practitioners, Medical Radiation Practitioners, Medical Practitioners, Nurses, Midwives, Occupational Therapists, Optometrists, Osteopaths, Paramedics, Pharmacists, Physiotherapists, Podiatrists and Psychologists
	National Alliance of Self Regulating Health Professions (NASRHP)	Audiologists, Dietitians, Exercise Physiologists, Speech Pathologists, Social Workers, Orthotists/Prosthetists, Perfusionists, Music Therapists and Genetic Counsellors
UK	Health and Care Professions Council	Arts Therapists, Biomedical Scientists, Chiropodists/Podiatrists, Clinical Scientists, Dietitians, Hearing Aid Dispensers, Occupational Therapists, Operating Department Practitioners, Orthoptists, Paramedics, Physiotherapists, Practitioner Psychologists, Prosthetics/Orthotics, Radiographers and Speech and Language Therapists
	Accredited Registers	Acupuncture, Alexander Technique, Aromatherapy, Audiology, Bioinformatics, Biomechanical Engineering, Biomedical Science, Botulinum toxins, Bowen Therapy, Cardiac Physiology, Chemical peels and skin rejuvenation, Children’s Health, Clinical Physiology, Clinical Technology, Complementary Therapies, Cosmetic Practitioners (Non-surgical), Counselling, Craniosacral Therapy, Dermal fillers, Foot Health, Gastroenterology Physiology, Hematology, Hair Restoration, Healing, Health Informatics, Healthcare Chaplaincy, Healthcare Science, Hypnotherapy, Injectable Cosmetic Providers, Kinesiology, Lasers, Intense Pulsed Light and Light-emitting Diode treatments, Life Sciences, Massage Therapy, Medical Engineering, Medical Illustration, Microbiology, Microsystems Acupuncture, Naturopathy, Neurophysiology, Nuclear Medicine, Nutritional Therapy, Physical Sciences, Physiological Sciences, Play Therapy, Psychotherapy, Public Health, Radiation Engineering, Radiation Physics, Radiotherapy Physics, Reflexology, Rehabilitation Engineering, Reiki, Renal Technology, Respiratory Physiology, Shiatsu, Sleep Physiology, Sport Rehabilitation, Sports Massage, Sports Therapy, Talking Therapy, Vision Habilitation, Vision Rehabilitation and Yoga Therapy

In Australia, the National Registration and Accreditation Scheme (NRAS) for health professions regulates 16 professions (Table 4). In addition, the National Alliance of Self Regulating Health Professions (NASRHP) a formal independent body aims to facilitate national consistency in quality, support for self-regulating health professions, and satisfy national and jurisdictional regulatory requirements. Nonetheless, considering the differences in jurisdictions among the different states and federal territories in Australia, minor variations in the list of regulated and unregistered allied health workforce prevailed. As for the professions that were neither regulated nor accredited by the NRAS, a range of laws was still applicable to regulate the practice. The clusters of law included the health complaint laws, regulation of threats to the public health (e.g., infectious diseases), consumer protection and employment laws as well as other relevant laws (e.g. criminal law, tort (negligence) and the law of contracts).

In the UK, the Health and Care Professions Council regulates 15 professions (Table 4). In the context of self-regulation, the system in the UK classified the professions into Accredited Registers (Table 4).

## Focus Group Discussion

Table 5 represents the codes and its frequency, as well as short descriptions of criteria for identifying PAH used in this present research.

**Table 5:** Codes and frequency, as well as short descriptions of criteria for identifying PAH

**No.**	**Codes**	**Description**	**Frequency**
(a) The more frequent codes			
1.	Risk of harm	Risk of inflicting harm to the practice and patient/client	62
2.	Set of competency & skills	Possessing the relevant competency and skill for healthcare system	46
3.	Formal Qualification	Highest relevant tertiary education attained by the practitioners (that are recognised or at least equivalent to the recognised ones in Malaysia)	40
4.	Defined scope of practice	The full spectrum of roles, functions, responsibilities, activities, and decision-making capacity that individuals within that profession are educated, competent, and authorized to perform	37
5.	Relevant training	A practitioner has undergone formalised and structured training modules as the requirement for attaining an academic qualification and fulfilling the professional requirement for certification	36
6.	Healthcare team	Practitioners that are involved as part of multi-disciplinary healthcare services.	18
(b) The least frequent codes			
1.	Related to people’s health	Direct/indirectly involved with people’s health such as preventive, promotion, rehab, therapy, palliative	8
2.	Autonomy in practice	Issues related to working independently	7
3.	Continuous professional development	Having specific professional development requirement	7
4.	Availability of code of conduct/ethics	Whether a profession or organisation has set of code of conduct/ethics in place	7
5.	Health-practicing certificate	Possessing valid Practicing Certificate	7
6.	Professional organisation / association	Qualified to register with specific organisation/association	5
7.	Career Pathway	A profession should have a career pathway from beginning till the end	4
8.	Rate of charges	The number of charges/fees set for their services	2
9.	Availability of a profession	Whether the profession is available across sectors or only within certain settings.	2
10.	International benchmarking	Comparison to what is happening oversea/worldwide	1

### Criteria to be considered as PAH

Sixteen codes were derived from the FGDs, with *risk of harm, set of competency and skills, formal qualification, defined scope of practice, relevant training* and *healthcare team* being the six most frequent codes (by hierarchy) as indicated by participants (Table 5 a). As for the remaining 10 codes, they were mentioned less than nine times during all the six FGDs cumulatively, with *related to people’s health* being the most frequently mentioned code within this category (Table 5 b).

#### Risk of harm

Risk of harm (for both patients and practitioners) was ranked as the most important criterion for determining PAH (frequency: 62), and the relevant quotations made by participants for supporting such a criterion focusing on patients are provided below:

**Table:** 

Participant ID	Stakeholder	Quotations
10	HEP	*“… when they finished their study, they're actually qualified to practice as a physiotherapist in any clinics or hospitals that provide rehabilitation therapy… if their skill or their treatment is not right and the diagnostic of this patient condition is not right; it (would) directly actually impact the process of recovery and also treatment of the patients”*
12	HEP	*“Those professions where you are directly handling the patients will perhaps have a higher risk of harm, because there are fewer of these levels of check and balance... (Since) those that are mainly lab-based, perhaps because of all these safety measures, will have less risk to the patient as opposed to those where you're directly intervening into the patient's care”*
14	Association	*“Because depending on the work that we do, which are patient-fronting, so there is a risk because it involves the patient’s well-being”*
20	Employer	*“As long as they qualify as allied health (practitioners) they will have some element of risk and mitigating that risk should be one of the objectives of the act”*
5	Regulatory Body	*“We are talking about public interest. So yes, because in this group it’s regulatory people, so safety of the patient comes first”*

In this context, the opinion of participants from the six different categories concurred that the risk of harm towards patients should be considered since the practitioners are directly involved with patient-handling (patient-fronting), and of public interest. Specifically, inaccurate diagnosis and treatment provided by the practitioner would result in the negative implications towards the recovery and well-being of the patients. In fact, efforts to mitigate the risk towards patient is one of the objectives in Act 774. It was also construed that practitioners providing medical/health laboratory services would exert lesser risk to the patients due to the presence of safety measures and their indirect involvement in patient care.

While the services provided by the practitioners shall be envisaged as *bona fide*, it is also important that the risk of harm exposed to the practitioners, while providing the services, is manageable. In this context, it can be seen that most of the participants agreed to the sentiment, the relevant quotations of which are provided below:

**Table:** 

Participant ID	Stakeholder	Quotations
2	HEP	*“They have been trained to anticipate of the risk and harm so that’s why I mentioned earlier, before you start delivering your services, your care and so forth, you should know… what are the red flags, something that you are not allowed to do. And the yellow flags you are allowed to do, but, maybe (with) extra caution. So, everything has been taught during their university life; all this kind of what… they do harm and risk and so forth”*
13	Association	*“The risk of not having it regulated is misinterpreted and that leads on to patients or individuals using the information wrongly and they take actions (against the practitioners)”*
1	Employer	*“Most of it is work place injury, especially those working in laboratories, because they are dealing with all the chemicals, harsh chemicals. And also (we) have problem with… now COVID pandemic, because most of our front liners are exposed to the disease… that’s the risk we are facing every day, … we also have problem with patients attacking our staff from time to time, especially in the psychiatric ward”*

The main issue raised by the participants for the harm to the practitioners, was learning to identify the risks and their management, which should be incorporated in academic syllabus as well as operational processes. Risk of harm on practitioners can also be associated with medico-legal actions (criminal and civil cases) in an instance malpractice or misconduct can be proven. Another important aspect that relates to the harm to the practitioners is the workplace; whereby practitioners can be exposed to harmful chemicals, contagious diseases, as well as physical assaults by patients/clients.

#### Set of competency and skills

The second most important criterion for describing PAH, as suggested by participants, is having a set of competency and skills (frequency:46). This would include undergoing sufficient hours of academic training and quick clinical experience, having the skill to perform the tasks (assessments, development of treatment plan and clinical reasoning) and ability to engage with patients, as well as attaining certain level of credential and competency. The followings are the relevant quotations made by participants for accentuating the paramount importance of such characteristics for defining PAH:

**Table:** 

Participant ID	Stakeholder	Quotations
2	HEP	*“Those who are dealing with health sciences should be qualified, undergone some training, some fairly quick clinical experience … can deliver, can engage with the public or patient ...”*
15	Association	*“We are being trained to understand why do I choose such an assessment tool? Or why do I decide on that assessment pathway? And then when it comes to developing the treatment plan, why does this person would benefit from such (a) treatment? The reason for the clinical reasoning is I feel … connecting the dots (of competency and skills)”*
17	Employer	*“The physiotherapist who see the patients need to have standards and the ability to also discuss with the patients what are the best options to rehabilitate them… the credential is required… making sure that certain hours (of training) have been accomplished during their study time”*
6	Regulatory Body	*“So the importance of registration is to ensure that all medical assistants working in government or private sectors are competent enough”*

#### Formal qualification

Formal qualification was deemed as the next important criterion for defining PAH (frequency: 40), and the pertinent statements made by participants, in this context, are provided below:

**Table:** 

Participant ID	Stakeholder	Quotations
12	HEP	*“To see whether their program of study is equivalent to what we have, or what we recognise within the country, right? We look at whether their qualifying, or their permissive exams, are something that we recognise, or at least are similar to what we do. So, those actually have to be done on an individual basis…”*
13	Association	*“… is the program recognised by the Public Service Department to practice the profession. That must be clear”*
17	Employer	*“To be recognised… they must have some basic qualifications aligned with the scope of their profession”*
6	Regulatory Body	*“Every profession should have their (own) set of criteria; what are the levels of education, what are the degrees involved for the profession? All these facts must be set as criteria for every profession. That is how we can register them”*

Participants were chiefly concerned about the fact that a practitioner must have a recognised academic qualification or at least, the qualification that is equivalent to the recognised ones in Malaysia, aligned with the scope of practice. Importantly, every profession shall require specific degree qualification as well as varying levels of education.

#### Defined scope of practice and healthcare team

The next two important criteria for describing PAH relate to having the specific scope of practice (frequency: 37) and allied health professionals working within a healthcare team (frequency: 18). Most participants advocated on having clear role and responsibility, scope of practice and the existence of Act 774 in providing a sense of security. The relevant quotations for supporting the defined scope of practice are provided below:

**Table:** 

Participant ID	Stakeholder	Quotations
2	HEP	*“I think the roles and also the responsibility should be clear”*
21	Association	*“The scope of practice must really clearly state (the) job, the job scope must be clearly stated”*
17	Employer	*“With Allied Health Professions Act, we have clearly distinguished between what are the profession scopes per say, what they can practice (and) what they could not, and it gives some sense of security, because if any medico-legal cases come up in the future, there is some reference that we can use to protect (the) organisations”*
3	Regulatory Body	*“We as a team, sometimes I would say there's some sort of overlapping, but you must know your boundaries”*

It must also be acknowledged that the field of medicine and healthcare can be overlapping, necessitating the importance of identifying the relevant boundaries of practice. The results of the FGDs also revealed that professionals working within the healthcare team is another defining criterion for PAH. The participants emphasised that to provide a holistic approach, multidisciplinary team input is pertinent. In this context, practitioners must have awareness about the scope of practice for other professions in order for their expertise to be utilised in the most suitable manner and instances. These two related aspects are accentuated in the following quotations:

**Table:** 

Participant ID	Stakeholder	Quotations
22	HEP	*“It is important for multidisciplinary team input. … we need a multidisciplinary input because patient(care) is a very holistic (approach)”*
16	Association	*“… for our profession, we will work with the patient and also several allied health professions such as physiotherapists, occupational therapists, and of course, the rehab physician and also sometimes the social worker.”*
19	Employer	*“that's why every week we have this discussion, of course, to actually educate each other about our scope of practice, because we realize that the gap is that we actually don't really understand other professions scope of practice. So that's why it becomes very difficult to refer and very, very difficult to actually know when and… how to refer.”*

#### Relevant training

Relevant training for the profession has been identified as equally important as it relates to the formal qualification and competency (frequency: 36). Suitable quotations from the participants representing HEPs, association, employer and regulatory body are provided below:

**Table:** 

Participant ID	Stakeholder	Quotations
12	HEP	*“… they must have this formalised, structured training that fulfils curricular requirements from MQA”*
16	Association	*“If they want to practice a new profession or a new career, as long as they get the right training, as long as they get the right certification to practice… I'm very okay with that”*
19	Employer	*“… for dietitians, we need (a) 800-hours of clinical placement, you know, within their degree…”*
5	Regulatory Body	*“… even (so,) for you to sit for the exam, we have to look at the training whether it's at par with our standard”*

It was evident that participants were concerned about PAH practitioners having formalised and structured training that is not only fulfilling the requirement of the profession but also the academic qualification agency. It was also mentioned that the training must be certified by the relevant authority, as well as having the minimum duration for clinical placement that would make it at par with the standard of the regulatory body for registration. As for the non-clinical PAH, undergoing industrial training, as recommended by the requirement of MQA, is necessary to ensure suitable competency.

#### Least frequently mentioned criteria

There are 10 additional codes for defining PAH identified through the FGDs namely *related to people’s health*, *autonomy in practice*, CPD, *availability of code of conduct/ethics*, *health-practicing certificate*, *professional organisation/association*, *career pathway*, *rate of charges*, *availability of a profession*, and *international benchmarking*. However, except for the ‘*related to people’s health*’ (frequency: eight), the frequencies for the remaining nine criteria were mentioned between one to seven times only during the FGDs.

Among the 10 codes, the “*related to people’s health”* had the highest frequency. The participants emphasised on the fact that PAH deals with patient’s assessment and intervention as well as related to healthcare services. Indeed, preventing from causing any harm to patient/client must be duly considered when providing assessment and intervention. The relevant quotations for supporting this particular criterion are provided below.

**Table:** 

Participant ID	Stakeholder	Quotations
2	HEP	*“… to assess the patient or the client whatsoever. And number two, they should be able to introduce some intervention.”*
16	Association	*“… of course, this profession deals directly with the patient… this profession involves assessment of the patient itself”*
20	Employer	*“… because allied health is related to healthcare. And we should follow the first act of healthcare, do no harm”*

Table 6 summaries the findings during the FGDs and the findings have been mapped with document review. The findings has been characterised according to criteria.

**Table 6:** Mapping of findings between document review and focus group discussions

Main Findings		Criteria
Document Review	FGDs	
Occupational safety and health in public health emergencies: a manual for protecting health workers and responders’ published by the WHO and International Labour Organization (ILO)(2018)	Risk of inflicting harm to patient and practitioners	Risk of harm
Role of association in defining and maintaining standards, aims and objectives and communications	Self-regulation and development of code of practice ethics by professions	
Regulations for PAH including the establishment of regulatory bodies in Malaysia, Australia and the UK.	The need for regulation to protect both public and practitioners, and for quality of services	
Malaysian Qualification Agency (MQA) released the latest version of Malaysian Qualification Framework version 2.0 (MQF 2.0) focusing on the relevant graduate attributes, program educational outcomes, program learning outcomes, course learning outcomes and constructive alignment Ministry of Higher Education (MOHE) published Framing Malaysian Higher Education 4.0: Future-Proof Talents depicting the aspirations, objectives, and approach to produce graduates that are capable to deal with artificial intelligence, digitisation, automation, and Internet of Things (IoT) in daily experience	Having relevant competency and skill for healthcare system; Having the highest relevant tertiary education possessed by practitioners Practitioners undergone formalised and structured training modules as requirement for attaining an academic qualification and fulfill professional requirement for certification	Set of competency and skills; Relevant training; Formal qualification
Different government entities are involved which it can be seen that the name of profession as well as the job scope remains generally similar.	Full spectrum of roles, functions, responsibilities, activities and decision-making capacity that individuals within the profession are educated, competent and authorised to perform Practitioner involved as a part of multi-disciplinary healthcare service.	Scope of Practice; Healthcare team

## Discussion

This FGD methodological technique is not only capable of bringing forth knowledge and information, but also an engaging consequential exploration of communication through perspectives, exposures, rich concepts, and opinions of participants [20]. In recent years, the application of virtual FGD in research is emerging and well suited particularly during pandemic COVID-19 [20–22]. Similarly, the FGDs for our research were conducted *via* the virtual platform to facilitate data collection from participants of various locations due to the movement control order imposed in Malaysia. The application of online platforms has been also reported by other previous studies [23–26], emphasising on similar and sound qualitative approach with specific protocols. The added advantage of virtual FGD is allowing constructive feelings of comfort as the FGD was conducted at the participants’ preferred venue, resulting in their dynamic involvement with robust content and pragmatic substance, despite diverse conflictual agreements [22].

The healthcare jurisdictions in Malaysia are uniform but effort must be made to clearly define the two important professions (*viz.* PAH and AHP) in the Malaysian healthcare framework for acknowledging the dynamic changes and embracing the importance of allied health services. The Act 774 has uniquely categorised the professions into AHPs and PAHs to provide a greater flexibility in reforming the healthcare workforce echoing to the notion advocated by international organisations and governmental agencies. However, the suitable criteria for defining PAH are not explicitly indicated in the Malaysian law. Thus, specific development in the regulation of AHPs and PAH in Malaysia appears nascent.

The present research that explored suitable criteria for defining PAH is timely, as it would support the successful implementation of Act 774 in the context of safeguarding the public interest and confidence in the overall framework of the healthcare system as well as promoting professionalism in the practice. While a review of the literature reveals the common utilisation of AHP terminology and its associated criteria for defining the profession, similar work for PAH is lacking. In this context, this inaugural attempt to define PAH proves necessary, considering the specific mention of such professions in Act 774. Results obtained through the FGDs unveiled six pivotal criteria for defining PAHs in Malaysia *viz.* risk of harm, set of competencies and skills, formal qualification, defined scope of practice, relevant training and healthcare team.

### Risk of Harm

Risk of harm being the most frequently mentioned criterion during the FGDs, its relevance to public safety has become the utmost priority in healthcare services although defining the risk of harm in healthcare settings can be challenging. Harm can also be implied as adverse events that consist of an unintended injury, injury causing prolongation of hospital stay, temporary/permanent disability or death, and injury caused by the healthcare management rather than the patient’s disease [27].

The participants in this study concurred that practitioners who are at risk of causing harm (directly and indirectly) must adhere to the standard protocols to avoid and mitigate such a possible harm when dealing with patients/clients. This is consistent with the indications made by Pham et. al. (2014), focusing on three prong aspects when measuring patient safety in the emergency department. The authors accentuated the need to evaluate the effectiveness of the existing policy for mitigating harms and the possible changes required to the policy for preventing future incidents. The subsequent step involves staff awareness assessment towards the existing policy and the changes made including measuring the effectiveness of its implementation through direct observations.

The participants also concurred that the potential harms may implicate the practitioners themselves in discharging their responsibilities. This would include medico-legal actions, contagious diseases, and physical assaults. As medico-legal actions relating to medical errors are invariably complex, the cases are generally revolved around inappropriate deviations by the practitioners from the best practice [28]. In this context, continuous education proves pertinent to equip the practitioners with the current development in providing service. This can be achieved by mutual engagement of stakeholders in developing suitable risk management curricula for the HEPs. This, in fact, is consistent with the findings from the document review analysis, specifically the MQF 2.0 document by the MQA for curriculum development and accreditation. The involvement of stakeholders is necessary, particularly for assessing the relevance and quality of the curriculum structure, including aspects pertaining to risks and harms. With regard to physical assaults (especially in the psychiatric facility), previous researchers emphasised the management commitment and employees’ involvement in safety health programs as the way forward to minimise the risks of physical assaults experienced by the practitioners [29]. Therefore, involvements of HEPs shall also be capitalised as suggested by the FGD participants to incorporate effective operational risk management in the academic syllabus and to enhance the understanding of the risk of harm imposed on both patients and practitioners. Triangulating this important finding with document review analysis on occupational safety and health by the WHO, ILO and, the Department of Occupational Safety and Health Malaysia in ensuring safety and/or minimising risk at the workplace and having periodical audits on such aspect, prove pertinent.

### Set of skills and competency

The second most frequently mentioned criterion for defining PAH is practitioners must have competencies and skills to perform their tasks. In addition, the FGD participants also stressed on having suitable clinical training hours to ensure adequate competencies, especially the skills of decision-making based on clinical reasoning in providing accurate patient assessment and treatment plan. Ladyshewsky et al. (2010) support this finding, whereby utilisation of hypothetical-deductive reasoning techniques in deciding clinical practice plan is imperative for achieving the optimum treatment outcome. Kurtz et al. (2018) indicates that competencies comprised of knowledge, technical, cultural, and communication aspects has widened the contextual relevance of skills and competencies for PAH. Considering the dynamic changes in the healthcare systems worldwide, transformative scale-up of skills and competencies among PAH are found paramount to support the needs of the country in providing up to date healthcare services. This is consistent with the recommendation by the WHO (2011) that the skills acquired during professional education requires upscaling to match the actual workplace requirements and epidemiology of the communities that they serve. Hence, the collaboration between the education, health sectors and other related authorities need to be strengthened to embrace the realities of health service delivery.

Several FGD participants in the research have also suggested the registration of PAH as a mean to determine standard for competency to practice and making compulsory CPD programs as part of upskilling efforts. Importantly, the overall findings from the reported FGDs are consistent with the document review analysis on HEPs and employers. As such, the findings supported the evidentiary value for skills and competencies as one of the suitable criteria for defining PAH in Malaysia. The focus on the attainment of competencies and skills through life-long learning as a means to improve quality of care and minimise risk of harms would demand clear governance framework for allied health practices. In particular, suitable competencies and skills for the management of prevention actions for non-communicable diseases and emerging communicable diseases as well as embracing new technology advancement in healthcare delivery must be enhanced.

In the era of the fourth industrial revolution, the fusion of physical, digital, and biological components is imminent in impacting the breadth of the different sectors [30] including healthcare. This is in line with the strategies outlined by the State of Queensland (2019) for optimising the allied health workforce for best care and value; outlining three strategic priorities such as adopting and digitally collecting standardised allied health data, utilising data analytics and implementing digitally enabled healthcare models by leveraging on advantages of technology. Similar approaches have been formulated in the 12^th^ Malaysia Plan to provide world-class healthcare services. Parallel to the strategic thrusts, the PAH services should embrace relevant digitalisation implementation plans for effective and responsive healthcare services, efficient and integrated care, and to improve service accessibility. Therefore, continuous improvement to the competency of healthcare professionals is undeniably vital.

### Formal qualification

Having formal specific tertiary qualification is deemed as an important criterion by the FGD participants for defining PAH despite the prevailing variability in the minimum qualifications acquired among PAH prior to practising, as stated in the Profile of Allied Health Professions in the MOH (2020). Currently, PAH needs to hold formal qualification from any local and international accredited programs endorsed by the MQA in order to register as AHP in Act 774. To be accredited, all academic programs in Malaysia must fulfil the stringent criteria of Accreditation, Equivalency and Standards Committees, using commonly agreed training competencies outlined in MQF 2.0 document published by the MQA (2017). Having a qualification agency to supervise and coordinate the academic quality and accreditation of higher education programs is also established in developed countries such as the USA [31], and the UK [32]. Taking into account the importance of formal qualification in defining PAH and AHP in Malaysia, AHSD has been working closely with relevant bodies particularly the MQA in listing the recognised qualifications and establishing the program standards.

In addition, collaborative efforts between the health and education sectors for providing inter-professional education models and credentialing for AHPs are imperative, as clearly demonstrated by state authorities in Australia (e.g., State of Queensland, 2019: State of Victoria, 2016) and the UK [32, 33]. Currently, the existing efforts in Malaysia between the MOH and MOHE to strengthen the curriculum contents periodically and to provide opportunities to practice is commendable. Periodical engagement sessions with relevant stakeholders including academics, employers, clinicians and accrediting bodies are recommended for improving the curriculum structure to meet the licensure and certification requirements, professional demand and societal needs [34–36]. The challenges are rather unique since the career pathways for several PAH and AHP are divided into two levels based on differences in formal qualifications *viz*. bachelor degree and diploma, indicating avenues for further improvements. If the relevant authority is planning to introduce single-tier pathway, a feasibility study to explore the issues, relevancy and the best model of implementation for PAH with minimum bachelor degree or higher qualification should be undertaken prior to policy establishment. In this regard, upgrading the diploma qualification to a bachelor degree or higher would enable competent autonomous practice among PAH in managing complex cases. Importantly, the academic programs offered by training colleges and universities should be fluidic in considering the changes in demographic and epidemiology, especially on issues relating to disease prevention, early diagnosis, early intervention and rehabilitation [37].

#### Defined scope of practice and healthcare team

Results from the FGDs further accentuated on having defined scope of practice and professionals working within a healthcare team as the next two important criteria for defining PAH in Malaysia. These findings appear consistent with those reported by other researchers [38, 39] focusing on various countries and/or jurisdictions. Frogner et. al. (2020) stated the dire need to ‘standardise evidence-based minimum scopes of practice for health professionals’ while allowing sufficient flexibility among the professions in rendering efficient and cohesive health services. Generally, workforce models in health have taken restricted approach specific to a profession with fixed scope of practice. However, recent models of workforce-planning have transformed into population-based approaches rather than profession-centred models [38]. Population based approaches has not only demonstrated positive outcomes for the public, but also the health professionals and optimisation of the health system [39] by ensuring all professionals are practicing to full scope [38] as prevailed during the COVID-19 pandemic globally.

Inadvertently as the AHP scope of services is evolving in the health care system in Malaysia, the professional boundaries in clinical practice need to be strategically addressed. For example, in the field of cardiothoracic surgery team of intensivists, perfusionists, medical assistants and AHPs such as physiotherapists, rehabilitation therapists, dietitians and social workers; each profession have inter-related scope of practice to ensure successful outcome [37]. Previous studies suggested improved outcomes in patients with diabetes mellitus [40, 41], anxiety and depression [42] as well as other clinical conditions [43] following multidisciplinary intervention. The participants concurred with the interrelatedness of the professions within medicine and healthcare in providing the best care in acute, chronic diseases and disorders management. Positive effects of working as a team includes better care continuity and coordination, beneficial changes in patient behaviour, improvement of patient symptoms and satisfaction [42, 44, 45]. Such inter-profession role may resulted in effective multidisciplinary care in community-based treatment and rehabilitation.

In addition to the multidisciplinary care, the service delivered by a single profession for special need remains crucial and widely accepted by medical fraternity. Practitioners would require to undergo certification programs before they can be entrusted to perform specialised scope of practice. However, recognising the relevant boundaries among the professions is still imperative, and its implementation may be challenging due to lack of awareness on the roles and skills of practitioners. This particular concern can be further exacerbated by having the fear of losing professional scope and job attributes, as well as the lack of common vision and goals [46–49]. In order to fortify allied health in Malaysia, it is vital to differentiate their respective scopes of practice to enhance structures that facilitate their expansion and integration of role into the health system. As the matter of fact, the current trend in healthcare services in the European countries and the UK has evolved towards expanding scopes of practice especially among nurses for performing specialised tasks that would require highly advanced training [50]. Such approach is made in view of limited number of expert professionals to accommodate the ever-escalating demographic and community progressions [51].

However, this skill-mix and changing the professional boundaries between the medical and allied health in a multidisciplinary team warrant a clear policy decision and awareness to address concerns when regulating a profession specific to their core scope of practice. In this regard, Nelson et al. (2014) indicated that the regulation of the scopes of practice involve achieving the equilibrium and flexibility among the intersecting dimensions to meet the need of the community and establish accountability among the different practitioners. There are however, inconsistencies in nomenclature and scopes of practice has been identified in the document review analysis between the public and private sector of PAH employers in Malaysia, which directly related to accountability. Therefore, standardisation of the nomenclature referring to ISCO-08 and scopes of practice of PAHs in Malaysia would provide a better platform for international data exchange, accreditation and transfer of expertise, while minimising unwanted confusions by the public. Specific studies to evaluate the appropriateness of having a common definition of scope of practice that transcends across different jurisdictions prove imperative. Reconciliation efforts are required to tackle sensitive issues relating to discrepancies in the source of powers, prerogative for assessing the competency, expanded scope of practice, and validation of CPD.

#### Relevant training

The fifth most commonly mentioned criterion was the relevant training. This refers to formalised and structured training modules as the requirement for both attaining an academic qualification and professional certification. In this context, there are two minimum requirement to practice, namely the relevance of the training and its minimum period, which may differ according to the varying professions. In Malaysia, it is not compulsory for allied health graduates to undertake housemanship, as compared to the medical doctor, dentist and pharmacist who are deemed required to complete stipulated period of housemanship [52–54]. However, the academic programs for allied health incorporated the compulsory industrial/clinical training within the academic programs as such component is crucial in the development of students’ skills and experience exposure at actual working settings. Currently MQA requires HEPs to allocate the number of credits based on the formula of one credit equivalent to 40 hours of training. For example, to graduate with the bachelor degree in occupational therapy, students are required to complete 30 to 44 credits (1200 to 1760 hours) of industrial training at relevant institutions [52–55]. For those in clinical programs such as dietetics, 800 hours of clinical training is required for one to graduate, apart from passing all the core and elective courses of the academic program [55]. This condition is made compulsory for the PAHs and AHPs to equip them with the relevant professional ethics as well as knowledge and skills that synchronise with the availability of facilities, competency setting and assessment [56–58], which are highly critical in performing their tasks. As a result of feasible and effective curricula, new allied health graduates are expected to be well prepared to adapt with the contemporary practice needs, as well as adjusting to the constantly challenging national and global health landscapes [36, 59].

An important concern found in the research was the practice of a practitioner outside of his field of expertise. For example, an optometrist performing embeded corneal foreign bodies removal at primary care without having the recognised training in such procedure. In this context, whilst having a full-fledged academic qualification in optometry is mandatory, the practitioner may consider undergoing relevant training and/or specific micro-credential programs to be recognised by the relevant authority. Acknowledging this current development in continuous education in Malaysia, the MQA has outlined suitable guidelines for HEPs to embrace micro-credential practice [60] although the adaptability of such an approach among the governing entities as well as stakeholders remains at its infancy stage. However, strong regulation and governance are imperative to ensure practitioners hold recognised relevant training and practice within boundary.

#### Least frequently mentioned criteria

The least frequently mentioned criteria identified in the research are equally important for a profession in moving to the next level of professionalism and for providing effective service delivery, even though the occurrences are less frequent. All of the criteria are deemed inter-related and embedded in the 6 main criteria emerged from the FGDs. For instance, ‘related to people’s health’ is clearly referring to the scope of practice for the PAHs which certainly involves preventive, promotive, curative, rehabilitative, and palliative actions [61]. It is evident in Malaysia that PAH has direct and indirect involvements in people’s health at all levels of care and settings [37]. While practitioners such as environmental health officers, entomologists and medical laboratory scientists are indirectly involved in people’s health, practitioners such as physiotherapists and clinical psychologists have direct involvements in patient’s care. For example, indirect involvements by the environmental health officers include surveillance and enforcement have become substantially demonstrated during the COVID-19 pandemic era, necessitating their important roles behind the scene.

The shift of healthcare paradigm and numerous technology breakthroughs have also altered the professionalisation and approach related to people’s health. The WHO advocated a holistic and people-centred care to cater the health needs and expectations of people and communities at every level [62]. As compared to the typical hospital-based services, the involvement of allied health practitioners outside of hospital care is increasing in trend, either in the community, remote or offshore environment [63, 64]. Utilising allied health practitioners for the management of non-urgent medical conditions and symptoms such as in elderly care home may be of beneficial to enhance primary care support, nutrition and hydration, rehabilitation and reablement as well as end of life and dementia care [65]. Griffiths et al. (2022) documented that AHPs were employed to promote healthy behaviours at home, virtual services, industry, local authority, social care, schools, nurseries and wider community in the effort of preventing obesity among young children as demonstrated by structured weight management program. In addition, utilisation of telehealth in Malaysia has been growing in parallel to the information communications technology advancement in the healthcare industry. For example, tele-rehabilitation development has been enabling AHPs to deliver professional services via video conference, which includes voice therapy [66], remote monitoring for chronic conditions [67], and managing cognitive frailty among older adults [68].

The current research in pursuit of defining the PAH add some values to the initiatives related to improving stewardship of allied health as outlined in the “Strategic Plan of the Allied Health Sciences Division & Allied Health Professions MOH 2021-2025” [69]. Hence, it is pertinent for the healthcare managers, allied health leaders and practitioners to recognise the criteria of PAH beyond the traditional healthcare setting. Establishment of the defining criteria will be of benefit for policymakers with comprehensive understanding on each of the criteria. For example, complete evaluation and categorisation of level of harm that may arise from a profession are necessary for classifying the profession as a high, moderate or low risk. As such practitioners of moderate to high-risks profession needs more of strict legislation, and would require the appropriate mandatory training as well as skills competency programs with close supervision, prior to independent practice. This is in view of embracing the socio-demographic changes, population needs, new healthcare professions, continuous expansion of technology in healthcare; such as health analytics, digital health, and precision medicine.

## Limitations and future perspectives

Being exploratory and qualitative in nature, the emergent criteria derived from stakeholders’ exploration of ideas reported in this research are the first attempt to provide suitable criteria for defining PAH in Malaysia despite several limitations. The small number of participants and the non-inclusiveness of participants from several parts of Malaysia (e.g. Sabah and Sarawak) as well as variations experience among participants representing HEPs, employers, associations and regulatory bodies may influence the generalisation of the study to the global level. Whilst it is impossible to include a larger number of participants as the research was conducted during the pandemic, future endeavour may obtain comprehensive feedback by recruiting larger number of participants; especially from other geographical regions in Malaysia since the delivery of healthcare is relatively unique in terms of epidemiology, needs and demands.

The implications of the present research also warrant future exploration in the development of career pathway for allied health practitioners with postgraduate or specialised training in Malaysia. The involvement of public consultation *via* survey, and examining the various perspectives of the public on PAH services, professional scope of practice, and their understanding towards the PAH criteria such as risk of harm, competency, skills, qualification and training would add considerable values and relevance to the professions since people are the focal point of healthcare services. In this context, in-depth studies focusing on the recent transformations of the healthcare sector (e.g. technological innovations in care provision, alternative modes of service delivery as well as mobility and team-based care), aiming at reducing costs, improving resource management efficiency and providing better disease prevention, treatment and rehabilitation, merit special consideration.

## Conclusions

Malaysia, being the recent country in the Southeast Asia region to establish legislation for allied health practitioners under Act 774, the criteria for defining PAH derived from this qualitative research is a distinct breakthrough to the healthcare system and policy direction for allied health in the country. The six main criteria for defining PAH in the context of the Malaysian healthcare system reported are risk of harm, set of competency and skills, formal qualification, defined scope of practice, relevant training and professionals working within a healthcare team. Additionally, the other least frequently mentioned criteria also present relevant weightage in developing governance structure for PAH, particularly for emerging health care professions. While there was no single identified criterion that can define PAH fully, policymakers should consider the cohesive approach of integrating all the empirically identified six main and 10 less frequently mentioned criteria for defining the profession and determine the qualifications, competency standards and roles of PAH, at least in the Malaysian’s perspectives. This research also emphasises the need for operational risk management in academic curriculum, the establishment of recognised formal qualification and competency standards, defined job scope, autonomy in practice, the need for professional standards development and the strengthening of single-tier qualification entry level into the workforce for PAH. Essentially, a defined criterion and the presence of Act 774 collectively inculcate a sense of assurance among allied health practitioners, instilling trust and safety to the public receiving the services. Hence, this research’s findings are significant in improving the comprehension about PAH in Malaysia, which is useful for policymakers in consolidating the healthcare governance for formulating suitable legislations and related informed policies to strengthen the regulatory framework of allied health practitioners in the country.

### Supplementary Information

**Additional file 1.** 

## Data Availability

All data generated or analysed during this study are included in this published article.

## Abbreviations

AHP: Allied Health Profession
AHSD: Allied Health Sciences Division
CPD: continuous professional development
FGD: focus group discussion
HEP: higher education provider
ILO: International Labour Organization
IoT: Internet of Things
ISCO-08: International Standard Classification of Occupation
MAHPC: Malaysian Allied Health Professions Council
MDC: Malaysian Dental Council
MOH: Ministry of Health
MOHE: Ministry of Higher Education
MQA: Malaysian Qualification Agency
MQF 2.0: Malaysian Qualification Framework version 2.0
NRAS: National Registration and Accreditation Scheme
PAH: Profession of allied health
T&CM Council: Traditional and Complimentary Medicine Council
UK: United Kingdom
USA: United States of America
WHO: World Health Organization
