# Supplementary material for: Developing criteria for a profession to be considered as profession of allied health in Malaysia: a qualitative study from the Malaysian perspective
Source: BMC Health Serv Res. 2024 Feb 2;24:165. doi: 10.1186/s12913-024-10569-0 (PMC10835829; doi:10.1186/s12913-024-10569-0)
Supplement: Supplementary file 1 — Additional file 1. [file 12913_2024_10569_MOESM1_ESM.docx]

**Moderator Guide Questions for Focus Group Discussion**

Key points to generate general questions for all stakeholders

a) Impression towards Act 774

b) Understanding of terms of PAH and AHP in the Schedule 2

c) Similarities and Differences between PAH and AHP

d) Other professions can be considered as PAH in Malaysia

e) Other health profession such as medical doctor practicing allied health services

| **General Question** | 1.What is your understanding/knowledge about the Allied Health Professions Act 2016 (Act 774)? |
| --- | --- |
|  | 2.What do you understand by the term “professions of allied health (PAH) and “allied health professions (AHP)”?   1. Can you suggest any kind of job/occupation/career that can be classified as PAH? 2. Based on the suggestions given, please explain why do you think so (differences/similarities)? |
|  | 3.What do you think about a Medical Doctor (for example) practising profession of allied health like Music Therapy? |

**Specific Question according to FGD Groups**

Key points to generate specific questions for HEP academics

a) Programs considered as related to allied health at the institutions

b) Institutions effort to ensure allied health programs are consistent with the requirements of the stakeholders for example the MOH

c) Programs perceived as high and low risks to patients/clients, the types of risk and management

d) Relevance to regulate all PAH under the Act 774

e) Relevance for regulating low risk professions

f) Industries/sectors that employ graduates from programs related to allied health

| **Specific questions for HEP academics** | 1.What is your understanding/knowledge about the Allied Health Professions Act 2016 (Act 774)? |
| --- | --- |
|  | 2.What do you understand by the term “professions of allied health (PAH) and “allied health professions (AHP)”?   1. Can you suggest any kind of job/occupation/career that can be classified as PAH? 2. Based on the suggestions given, please explain why do you think so (differences/similarities)? |
|  | 3.What do you think about a Medical Doctor (for example) practising profession of allied health like Music Therapy? |

Key points to generate specific questions for health associations

a) Roles and functions of association in terms of regulation and professional conduct of members

b) Characteristics of PAH that should be regulated or would require statute regulations

c) Relevance to regulate all PAH under the Act 774

d) Self-regulation of PAH and its suitability in Malaysia

| **Specific questions for Health Associations** | 1. What are the scopes of practice for your profession in various practice setting? |
| --- | --- |
|  | 1. Please explain the importance of your professions in various setting? |
|  | 1. Would you consider your profession as inflicting a high and/or low risk to patients/clients? 2. Could you explain the harm/risk involved? 3. With regards to risk/harms, how does your association incorporate risk management measures? 4. If you considered as low risk, should your profession be regulated in any Act? Why? |

Key points to generate specific questions for employers

a) Types of PAH employed at the institutions

b) Criteria for recruiting PAH

c) Scopes of practice for PAH

d) PAH and the associated risk to patients/clients

e) Relevance to regulate all PAH under the Act 774

| **Specific questions for Employers** | 1. Please explain the importance of having allied health professions in your institution? |
| --- | --- |
|  | 1. What are the criteria for recruiting allied health practitioners? |
|  | 1. What are the scopes of practice for allied health practitioners in your setting? |
|  | 1. What is your opinion about allied health practitioners appointed for a specific job scope but also performing other allied health tasks? |
|  | 1. Among the allied health practitioners in your institutions which do you perceive as inflicting high and low risk to patients/clients?    1. Could you explain the harm/risk involved?    2. With regards to risk/harms, how does your institutions incorporate risk management?    3. If the practitioners are considered as low risk, should they be regulated in any Act? Why? |

Key points to generate specific questions for regulatory bodies

a) Functions/roles of the Regulatory Body

b) Criteria for recognizing a profession as PAH

c) Relevance of regulating all PAH under the Act 774

d) Characteristics of PAH that would require statute regulations

e) Characteristics of PAH that can be considered for other types of regulation (e.g. self-regulation and co-regulation etc.)

| **Specific questions for Regulatory Bodies** | 1. What are the functions/roles of your council? |
| --- | --- |
|  | 1. In your opinion, what are the criteria to be categorized as professions of allied health? |
|  | 1. Should we regulate all professions of allied health? |
|  | 1. What are the characteristics of professions of allied health that would require statute regulations? And why? |
|  | 1. In view of the current global trend of self-regulation among healthcare professions, what is your opinion about such practice and its’ suitability for practitioners in Malaysia? |

Key points to generate specific questions for heterogenous group

a) criteria to be categorized as professions of allied health?

b) Relevance of regulating all PAH under the Act 774

c) Scopes of practice for PAH

d) PAH in various settings and institutions

e) Harms/risks involved in the practice of allied health

| **Specific questions for Heterogeneous group** | 1. What is your opinion of health care personnel who practise/provide allied health related services? |
| --- | --- |
|  | 1. What the harms/risks involved in the practise of allied health? |
|  | 1. What is your opinion about the Allied Health Professions Act 2016 (Act 774)? |
|  | 1. Should we regulate all PAH? |
|  | 1. What are the characteristics of PAH that would require statute regulations? And why? |
|  | 1. In view of the current global trend of self-regulation among healthcare professions, what is your opinion about such practice and its’ suitability for practitioners in Malaysia? |
